# Do Haemodynamic Definitions of Chronic Thromboembolic Pulmonary Hypertension Distinguish between Distinct Phenotypes of Chronic Thromboembolic Pulmonary Disease?

**DOI:** 10.1513/AnnalsATS.202405-524OC

**Published:** 2025-03-01

**Authors:** Ellis Cerrone, Abdul G. Hameed, David G. Kiely, Robin Condliffe, Andrew J. Swift, Smitha Rajaram, Ian Smith, Judith A. Hurdman, Charlie A. Elliot, A. A. Roger Thompson, Alexander M.K. Rothman, Athanasios Charalampopoulos

**Affiliations:** 1 Sheffield Pulmonary Vascular Disease Unit, Royal Hallamshire Hospital, Sheffield, United Kingdom; 2 Department of Infection, Immunity and Cardiovascular Disease, University of Sheffield, Sheffield, United Kingdom; 3 INSIGNEO, Institute for Insilico Medicine, University of Sheffield, United Kingdom; 4 NIHR Sheffield Biomedical Research Centre, Sheffield, United Kingdom; 5 Department of Radiology, Sheffield Teaching Hospitals NHS Foundation Trust, Sheffield, United Kingdom

## Abstract

**Background:**

Chronic thromboembolic pulmonary disease (CTEPD) is defined by chronic organised thrombi in the pulmonary circulation without or with pulmonary hypertension (CTEPH). The current definition of CTEPH has adopted lower mean pulmonary artery pressure (mPAP) and pulmonary vascular resistance (PVR) thresholds with unclear impact on the characterisation of patients with CTEPD.

**Methods:**

All consecutive CTEPD patients referred for cardiopulmonary exercise testing (CPET) in a PH centre were divided into four groups based on pulmonary haemodynamics. Group 1: mPAP≤20 mmHg, Group 2: mPAP>20 mmHg with PVR>2 and ≤3 WU, Group 3: mPAP>20 mmHg with PVR>3 WU, Group 4: mPAP>20 mmHg with PVR<2 WU (“unclassified”). We compared CPET, CT pulmonary angiography, and MRI data across the groups.

**Results:**

There was mild aerobic capacity impairment, mild/moderate ventilatory inefficiency, and no significant cardiac limitation on CPET in all groups. However, patients in Groups 1 and 4 had better ventilatory efficiency and less oxygen desaturation on exercise due to lower dead-space ventilation. There was no difference in chronic pulmonary emboli burden and distribution, or resting RV function between the groups. Seventeen patients were reclassified as having “CTEPH” based on the current definition. No functional deterioration was noted within a median period of 13 months on repeat CPET.

**Conclusions:**

CTEPD patients with similar clot burden and RV function without or with mild/moderate PH displayed a similar pattern of cardiopulmonary limitation, except for ventilatory efficiency. The current definition for CTEPH may lead to reclassification of CTEPH in a considerable number of patients.

## Introduction

Partial resolution of pulmonary emboli (PE) after an acute event can lead to residual post-thrombotic changes in pulmonary circulation in at least 15-30% of all patients^1^. These changes consist of organised fibrotic blood clots causing proximal pulmonary artery (PA) stenosis or obstruction and/or microvasculopathy affecting vessels with a diameter <500 μm^2, 3^. These changes may also increase pulmonary arterial pressure and resistance leading to pulmonary hypertension (CTEPH) in ~3% of patients^4^.

Over the last few years, the haemodynamic definition of CTEPH has changed and the thresholds for both mean PA pressure (mPAP) and pulmonary vascular resistance (PVR) have lowered. In the 2015 ESC/ERS PH guidelines CTEPH was defined as mPAP≥25 mmHg, and PA wedge pressure (PAWP)≤15 mmHg, with radiographic evidence of organised PA thrombi after 3 months of anticoagulation^5^. In 2018, at the 6th World Symposium on PH (6^th^ WSPH), a new definition was suggested with mPAP>20 mmHg combined with a PVR≥3 Wood units (WU) and PAWP≤15 mmHg^6^. In the 2022 ESC/ERS PH guidelines CTEPH was defined as a form of pre-capillary PH with mPAP>20 mmHg and PVR>2 WU^7^.

A cohort of patients with chronic PE, exertional breathlessness, but no resting PH has been described^8, 9^. The term initially coined to distinguish this condition from CTEPH was ‘chronic thromboembolic disease’. In 2021 the broader term ‘chronic thromboembolic pulmonary disease’ (CTEPD) was proposed by expert consensus^10^. CTEPD describes all symptomatic patients with organised fibrotic clots and/or perfusion defects on imaging despite at least 3 months of anticoagulation who may or may not have PH at rest. If there is no PH, the disease is called ‘CTEPD without PH’, whereas if there is, the term CTEPH remains. This terminology was later adopted by the 2022 ESC/ERS guidelines.

In this study we examined a cohort of patients with CTEPD without and with PH who underwent cardiopulmonary exercise testing (CPET) at a PH referral centre. We sought to explore whether the different haemodynamic definitions for CTEPH would help differentiate patients with distinct characteristics with respect to exercise physiology, clot burden and distribution, and right ventricular (RV) function at rest.

## Materials and Methods

### Patients

We evaluated all consecutive CTEPD patients without or with PH and dyspnoea referred to the CPET lab of the Sheffield Pulmonary Vascular Disease Unit from 2019-2023 from the ASPIRE Registry^11^. Symptomatic patients with CTEPD without PH routinely undergo CPET in our institution for the impact of chronic clots on exercise capacity to be evaluated, whilst patients with CTEPH were referred for the major cause(s) of dyspnoea to be identified, when it could not be fully explained by pulmonary haemodynamics. Four comparison groups were formed: Group 1 consisted of patients with a mPAP≤20 mmHg, Group 2 mPAP>20 mmHg and PVR>2 and ≤3 WU, Group 3 mPAP>20 mmHg and PVR>3 WU, and Group 4 mPAP>20mmHg and PVR<2 WU, which is regarded as “unclassified” PH within the current definition. Baseline demographics, CPET, CT pulmonary angiography (CTPA), and cardiac magnetic resonance (MRI) data were compared between the groups.

Patients who demonstrated a significantly abnormal exercise response on the baseline CPET were followed up with a repeat test at the clinician’s discretion. The baseline and repeat tests were compared to assess for evidence of disease progression.

### Right Heart Catheterisation (RHC)

RHC was performed using standard procedures^12^ and mPAP, PAWP, cardiac output (CO), cardiac index (CI), PVR, and PA compliance were obtained. PA compliance was defined as stroke volume (SV)/pulmonary pulse pressure (PP), where SV was calculated as CO/heart rate, and PP as the difference between systolic and diastolic PA pressure. CO was measured using the thermodilution method.

### Cardiopulmonary exercise test (CPET)

Patients underwent CPET on an upright cycle ergometer using a breath-by-breath system (Vyntus CPX, Vyaire Medical, Germany) according to the American Thoracic Society/American Society of Chest Physicians statement on CPET^13^. The testing protocol and relevant equations are described in the Supplementary material. For each patient, we assessed the reason for exercise limitation on CPET. We used patterns described in previously published work^14^. The pattern of “cardiopulmonary limitation” was defined as VE/VCO_2_ slope ≥30, and/or a peak O_2_ pulse<80% predicted with a respiratory exchange ratio over 1.05. Limitation of exercise capacity due to “ventilatory exhaustion” was defined as a breathing reserve<15% of maximal voluntary ventilation, and oxygen desaturation at peak by at least 4%. “Normal cardiorespiratory response to exercise” was defined as peak VO_2_>80% predicted, VE/VCO_2_ <30, and peak O_2_ pulse>80% predicted.

### Imaging

All patients had undergone CTPA. The number of PA segments with chronic thromboembolic changes was retrieved from the reports produced by two expert Thoracic Radiologists after an MDT discussion. A qualitative assessment of scarring and mosaicism on CT was performed by the same Radiologists blinded to each other. Their inter-observer reliability was tested using Cohen’s Kappa.

All patients had also undergone MRI and RV ejection fraction (RVEF) was calculated using an established technique with high interobserver reproducibility^15^. RV-PA coupling is defined as the ratio of end-systolic RV elastance and effective arterial elastance, and was calculated on MRI as ESV/SV, where ESV is end-systolic RV volume^16^.

### Statistical analysis

Continuous variables were first assessed using the Shapiro-Wilk test of normality. Those with a normal distribution are presented as mean +/-standard deviation. Skewed data are presented as median (interquartile range). Categorical data are shown as frequency(%).

Continuous normally distributed variables were compared between the four groups using a one-way ANOVA. Skewed data were compared using the Kruskal-Wallis H test, whilst the comparison between categorical data was done using the χ^2^ test. For the repeat CPET analysis we compared average values of the baseline test with those of the repeat using the paired student’s t-test. Binary logistic regression was performed to assess the effect of various parameters on the odds of a patient having a VE/VCO_2_ slope>36 or a peak oxygen pulse<80% predicted. These cut-off limits represented significant cardiorespiratory limitation in previous studies^14^. Differences for all analyses were considered statistically significant when p value was <0.05. Statistical analysis was carried out using IBM’s SPSS statistics package, version 29.

### Ethics

This study was conducted using data extracted from the ASPIRE registry (research database REC ref 22/EE/0011). This study was approved by the ASPIRE data management committee and data were extracted and anonymised as per ASPIRE standard operating policy.

## Results

**Figure 1:**
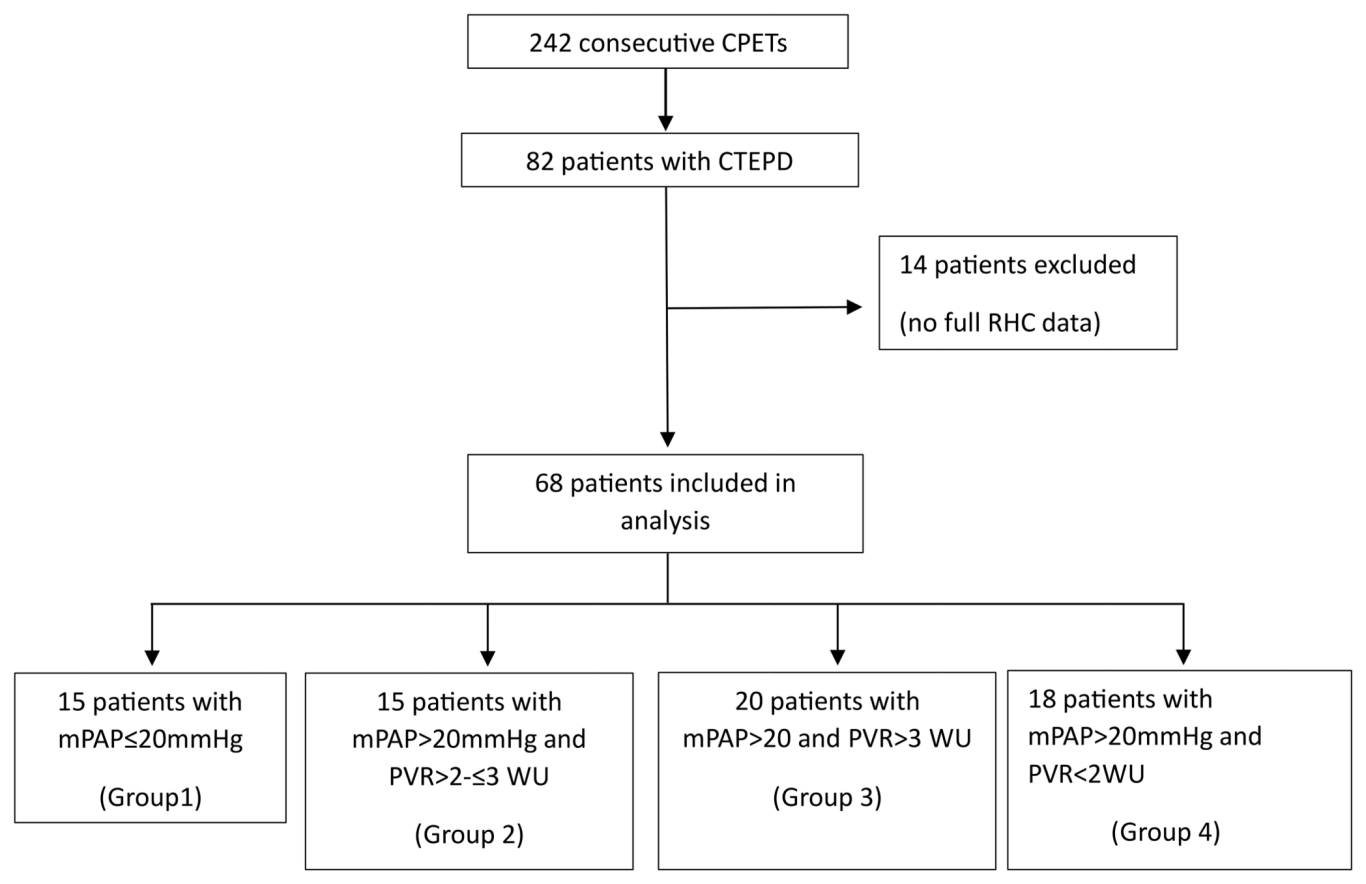
Consort diagram CPET, cardiopulmonary exercise test; CTEPD, chronic thromboembolic pulmonary disease; RHC, right heart catheterisation; mPAP, mean pulmonary artery pressure; PVR, pulmonary vascular resistance; WU, Wood units.

Fourteen of the total 82 patients were excluded due to lacking complete RHC data (Figure 1). Baseline characteristics are shown in Table 1. Group 2 met the 2022 ESC/ERS criteria for CTEPH, whilst Group 3 met both the 6^th^ WSPH and 2015 ESC/ERS definitions. There was no significant difference in age, sex, incremental walk test distance, or NT-proBNP, whist there was a trend towards a higher BMI in the “unclassified” PH group. The latter also had a higher PAWP and CO. Despite the higher PAWP, left atrial (LA) size on CTPA was within normal range (mean LA area: 18.5 +/- 4.8cm^2^) making latent PH due to left heart disease less likely. Group 3 had the lowest CO/CI of all groups, and a trend towards lower FEV1.

**Figure 2:**
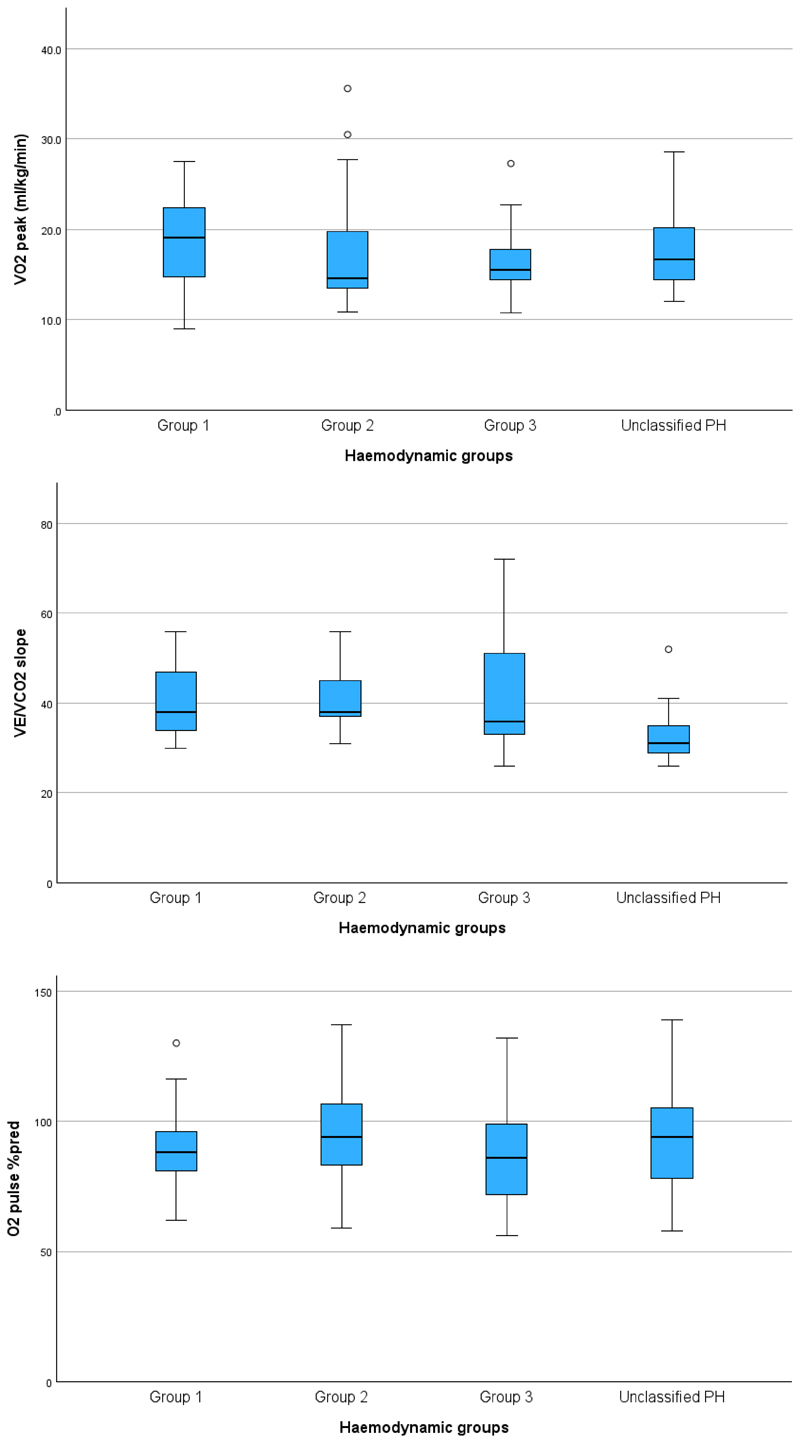
Graphical display of peak VO_2_ in ml/kg/min, VE/VCO_2_ and O_2_ pulse % predicted per haemodyamic group.

CPET results for each group are shown in Table 2 and Figure 2. Although, there was no difference in peak VO_2_, Group 4 had better ventilatory efficiency with a significantly higher partial pressure of end-tidal CO_2_ (P_ET_CO_2_) at anaerobic threshold, and a trend towards a lower VE/VCO_2_. In addition, Group 1 and 4 demonstrated higher arterial partial pressure of oxygen (PaO_2_) at peak, lower alveolar-arterial gradient (A-a gradient) and a trend for lower dead-space to tidal volume ratio (V_D_/V_T_) compared to the CTEPH groups. Interestingly, Group 4 showed an increase of peak oxygen saturation. All patients in this study showed evidence of “cardiopulmonary limitation”, whilst 5 also showed “ventilatory exhaustion” (one in Group 1, three in Group 2, and one in Group 3) as defined above. No patients had a “normal cardiopulmonary response”.

Imaging data are shown in Table 3. Inter-observer reliability between the two Radiologists for the assessment of scarring/mosaicism was substantial (Kappa=0.716). There was no statistically significant difference in mosaicism, scarring, clot distribution (proximal, segmental, distal), or the number of PA segments involved. There was a trend towards a lower RVEF in Group 3.

Logistic regression showed that none of the variables tested reached statistical significance in our cohort (Tables S1, S2).

Nineteen patients had a repeat CPET; two in Group 1, five in Group 2, eleven in Group 3, and one with “unclassified” PH. There was no change in medical treatment between the two tests. The median time from baseline to repeat test was 13 (11-21) months and there was no significant difference in any of the CPET parameters between the two (Table 4). Four patients died of causes unrelated to CTEPD (malignancy, pancreatitis), and one had pulmonary endarterectomy (PEA) within the period of our observation (4 years). In our cohort, 19 patients had “CTEPH” with the 2015 ESC/ERS, 20 with the 6^th^ WPHS and 36 with the 2022 ESC/ERS definition.

## Discussion

Our study explores the impact of different haemodynamic definitions of CTEPH on characterising patients with CTEPD. We show that: 1) patients with CTEPD without and with PH with a PVR up to 5 WU had mild reduction of aerobic capacity (peak VO_2_ %predicted), and no signs of significant cardiac limitation (peak O_2_ pulse %predicted) on CPET, 2) patients with “unclassified” PH showed the best ventilatory efficiency (highest P_ET_CO_2_ and trend towards lower VE/VCO_2_), whereas both the “CTEPD without PH” and the “unclassified” PH groups had less oxygen desaturation, and lower dead-space ventilation (lower A-a gradient) at peak compared to the other two groups, 3) there was no difference in chronic clot burden and distribution between the groups, 4) 17 patients were reclassified as having “CTEPH” based on the 2022 ESC/ERS definition compared to 2015, 5) no functional deterioration was observed within a median period of 13 months as assessed by repeat CPET.

Our CPET findings are consistent with previously published data. Swietlik et al^17^ showed similar range of peak VO_2_, O_2_ pulse, and VE/VCO_2_ in patients with CTEPD with mPAP<25 mmHg. Held et al^18^ showed that patients with CTEPH with a median PVR of 5.9 WU had a higher A-a gradient compared to those with mPAP<25 mmHg. McCabe et al^19^ and Claeys et al^20^ showed a lower peak VO_2_ along with a higher VE/VCO_2_ and dead-space ventilation in CTEPH patients compared to those with mPAP<25 mmHg; however, their CTEPH cohorts had more severe disease than ours. Our study is the first one to analyse a group of patients with mPAP>20 mmHg and PVR<2WU who have “unclassified” PH according to the 2022 ESC/ERS PH definition. These patients tend to have higher BMI, PAWP, and CO/CI, with better ventilatory efficiency than all other CTEPD groups, and as low dead-space ventilation and high peak PaO_2_ as “CTEPD without PH”. Hence, it appears that they behave similarly with those without PH and that their higher CO probably drives a higher mPAP. Higher CO may also explain their ability to eliminate CO_2_ more efficiently during exercise. However, more studies are needed to clarify whether they represent a distinct pathophysiological group or whether it is a weakness of the current CTEPH definition to separate them from “CTEPD without PH” where they may truly belong.

Claeys et al^20^ used exercise MRI to show that RVEF increased with exercise in patients with CTEPD without PH as opposed to CTEPH. However, when the former were compared to healthy controls, RV contractility and CO rose at a lower rate due to chronotropic incompetence and a blunted SV response. In addition, van Kan et al^21^ showed that 50% of patients with CTEPD without PH demonstrate a steep mPAP/CO slope> 3 mmHg.min.L^-1^ and low PA compliance on invasive CPET. With regards to RV-PA coupling, Douschan et al^22^ showed in a retrospective analysis that patients with CTEPD without PH had lower TAPSE/systolic PA pressure ratio than healthy individuals but higher than CTEPH. In our analysis we showed no difference in RV-PA coupling by using an MRI-derived validated index, but our CTEPH patients had milder haemodynamics. Overall, it appears that patients with “CTEPD without PH” are in the middle of a spectrum with healthy individuals on one end and CTEPH patients on the other in terms of their aerobic capacity, ventilatory efficiency and RV contractile reserve. The higher the PVR, the more pronounced functional limitation and ventilatory inefficiency due to dead-space ventilation would be. The question whether exercise invasive haemodynamics are more sensitive than CPET to elicit cardiovascular limitations in patients with CTEPD remains open.

Most of our patients had proximal/segmental chronic PE, but there was no difference in the number of PA segments involved or the extent of mosaicism and scarring on CT between patients without or with mild/moderate CTEPH. Reddy et al^23^ showed a higher macrovascular disease burden in patients with mPAP between 21-24 mmHg compared to ≤20 mmHg, but this difference did not reach statistical significance. Capone et al^24^ also showed no difference in the degree of vascular obstruction between patients with CTEPD without or with PH, but there was more mosaicism in their CTEPH cohort who had severe haemodynamics. The presence of modest mosaicism in our population could be explained by the presence of very little distal disease/microvasculopathy which is also reflected by a modestly elevated PVR. Reddy et al^23^ also examined the natural history of CTEPD patients with mPAP<25 mmHg and showed no difference in WHO functional class, echocardiographic parameters, NT pro-BNP, or 6-minute walk test distance at 36 months. In our cohort there was also no difference in CPET parameters within a median period of 13 months. Nevertheless, longer follow-up is required to establish whether CTEPD without PH is an early stage of CTEPH with slow progression or a separate disease that remains stable over time.

The optimal management of patients with CTEPD without or with mild PH remains unclear. PEA in patients with CTEPD without PH has led to significant improvement of exercise capacity and quality of life in small single-centred studies and selected patients^8,21,25^. More recently, Ghio et al^26^ confirmed this improvement in patients with mPAP between 21-24 mmHg (mean PVR 3.4 WU) but not in a very small group of patients with mPAP<20 mmHg and PVR<2 WU. In addition, complications related to PEA have been reported in up to 40%^8^. Hence, if patients with CTEPD without or with mild PH do not demonstrate severe functional impairment, and their disease does not progress over time, the need for a major intervention such as PEA would be less compelling. Balloon pulmonary angioplasty seems to be promising^27,28^, but larger studies are required to establish its value in these patients. If we take into consideration that 50% of patients after an acute PE remain symptomatic due to cardiopulmonary limitations^14^, the management of these patients, with or without CTEPD, will continue to pose a considerable challenge for physicians until more definitive data are available.

## Limitations

Our study is small, single-centred and retrospective in nature, and there might be a bias in the selection of patients with a PVR>3 WU, as not all patients with CTEPH undergo CPET in our institution. However, we included all consecutive patients with CTEPD referred for CPET in a national PH centre, and all patients were investigated based on a standardised protocol in terms of cardiothoracic imaging, RHC, and CPET. The small size of our cohort could raise the suspicion that we did not detect differences between the groups due to our study being underpowered. Although this might be true, our outcomes appear to be consistent with previous published ones. Our assessment of chronic PE burden was not performed by the modified Qanadli index, but by using another quantitative measure (number of PA segments involved). Mosaicism and scarring were assessed in a qualitative manner by two very experienced Radiologists blinded to each other, and with excellent inter-observer reliability. Not all our patients underwent a repeat CPET. This was mainly because patients with no major abnormalities on the baseline test were discharged. Furthermore, our follow-up time was short and therefore it is hard to draw safe conclusions about the natural history of the disease. Finally, our cohort consists of symptomatic patients with CTEPD, therefore we are unable to make any comments on asymptomatic patients with chronic PE.

## Conclusions

In CTEPD patients with similar clot burden and RV function, those without or with “unclassified” PH displayed a similar pattern of cardiopulmonary limitation, albeit better ventilatory efficiency, to those with mild/moderate CTEPH based on current haemodynamic definitions. The 2022 ESC/ERS haemodynamic definition of CTEPH may lead to reclassification of CTEPH in a considerable number of patients with unclear benefit. Larger prospective studies are required to confirm this finding.

## Supplementary Material

Supplementary Materials

## Figures and Tables

**Table 1:** Baseline characteristics

Variable	Group 1 mPAP≤20 mmHg n=15	Group 2 mPAP>20 mmHg and PVR >2-≤3 WU n=15	Group 3 mPAP>20 mmHg and PVR>3 WU n=20	Group 4 “Unclassified” mPAP>20mHg and PVR<2WU n=18	p value
Age (years)	51.3 (+/-15.3)	53.4 (+/-18.1)	60.6 (+/-12.6)	54.9 (+/-12.7)	0.271
Male	8 (57.1%)	10 (66.7%)	15 (75%)	11 (61.1%)	0.706
BMI	30.5 (+/-7.3)	30.2 (+/-5.5)	30.5 (+/-6.5)	35.3 (+/-6.3)	0.077
Hb (g/L)	156.2 (+/-18.8)	155.2 (+/-14)	168.7 (+/-17.3)	153.3 (+/-21.4)	0.184
NT-proBNP (pg/ml)	51 (20.5-151.5)	104 (66-210)	153 (45-480)	65 (34.5-117)	0.083
ISWT (m)	367 (+/-145)	521 (+/-263)	361 (+/-215)	407 (+/-222)	0.150
mPAP (mmHg)	18 (16.5-19.5)	26 (23.5-29)	30.5 (26-43)	23 (22-24.25)	<0.001
PAWP (mmHg)	7.9 (+/-3)	10.6 (+/-2.5)	10.2 (+/-3)	13.6 (+/-3.2)	<0.001
Cardiac output (L/min)	6.2 (+/-1.1)	6.1 (+/-0.9)	5.2 (+/-1)	6.8 (+/-1.3)	<0.001
Cardiac index (L/min/m^2^)	3.1 (+/-0.7)	3.1 (+/-0.7)	2.6 (+/-0.4)	3.2 (+/-0.6)	0.003
PVR (WU)	1.7 (+/-0.7)	2.6 (+/-0.3)	4.9 (+/-2.3)	1.5 (+/-0.3)	<0.001
PAC (L.mmHg^-1^.min^-1^)	4.2 (3-5.2)	2.9 (2.7-3.4)	2 (1.3-2.3)	3.9 (2.8-4.7)	<0.001
FEV1 (% pred.)	91.5 (+/-22)	79.3 (+/-18.5)	74.9 (+/-16.7)	84 (+/-12.7)	0.072
FVC (% pred.)	99.4 (+/-24.1)	87.1 (+/-16.3)	85.5 (+/-17.2)	89.9 (+/-12.1)	0.163
FEV1/FVC%	72.8 (+/-8)	71.7 (+/-7.3)	68.2 (+/-11)	74.7 (+/-9.6)	0.22

mPAP, mean pulmonary artery pressure; PVR, pulmonary vascular resistance; WU, Wood units; BMI, body mass index; Hb, haemoglobin; NT-proBNP, N-terminal pro-Brain Natriuretic Peptide; ISWT, incremental shuttle walk test; PAWP, pulmonary arterial wedge pressure; PAC, pulmonary artery compliance; FEV1, forced expiratory volume in 1 second; FVC, forced vital capacity.

**Table 2:** Cardiopulmonary Exercise Testing variables.

Variable	Group 1 mPAP≤20 mmHg n=15	Group 2 mPAP>20 mmHg and PVR >2-≤3 WU n=15	Group 3 mPAP>20 mmHg and PVR>3 WU n=20	Group 4 “Unclassified” mPAP>20mHg and PVR<2WU n=18	p value
Work (%pred.)	87.7 (+/-25.5)	89.7 (+/-29)	88 (+/-23.5)	90.8 (+/-25)	0.983
BR (% of MVV)	26.4 (17-42.6)	25.7 (8.4-39)	23.5 (15-42)	28.3 (17-42)	0.416
Peak VO_2_ (ml/kg/min)	18.7 (+/-5.5)	18.1 (+/-7.4)	16.5 (+/-4.2)	17.4 (+/-4.8)	0.718
Peak VO_2_ (% pred.)	73.3 (+/-14.8)	74.4 (+/-19)	74.8 (+/-15)	78.7 (+/-23)	0.846
RER peak	1.14 (+/-0.1)	1.13 (+/-0.1)	1.11 (+/-0.1)	1.14 (+/-0.1)	0.844
VO_2_/work	8.7 (+/-0.9)	8.8 (+/-1.2)	8.4 (+/-1.5)	8.8 (+/-1.4)	0.712
Peak O_2_ pulse (% pred.)	91 (+/-17.1)	96 (+/-21.5)	89 (+/-20.6)	93(+/-20.9)	0.768
VE/VCO_2_ nadir	38.5 (+/-7.9)	38.9 (+/-7.3)	37.4 (+/-7.4)	33 (+/-6)	0.075
VE/VCO_2_ slope	39.9 (+/-7.9)	40.4 (+/-7.5)	40.3 (+/-9.3)	33.2 (+/-6.3)	0.063
P_ET_CO_2_ at AT (kPa)	3.8 (+/-0.5)	3.6 (+/-0.7)	4 (+/-0.9)	4.4 (+/-0.6)	0.011	
ΔSO_2_ (%)	-2 (0-4)	-4 (1-8)	-4 (1.5-6)	3 (1-5.7)	0.021
Peak PaO_2_ (kPa)	11.7 (+/-2.3)	9.6 (+/-2.8)	9.3 (+/-2.1)	11.7 (+/-1.5)	0.015
Peak lactate (mmol/L)	6.6 (+/-2.8)	5.9 (+/-2.4)	6.3 (+/-2.5)	6.6 (+/-2.2)	0.923
A-a gradient	3.8 (+/-2.5)	6 (+/- 2.4)	6 (+/-2.2)	3.7 (+/-1.4)	0.014
V_D_/V_T_	0.35 (+/-0.1)	0.41 (+/-0.1)	0.39 (+/-0.2)	0.29 (+/-0.1)	0.067

mPAP, mean pulmonary artery pressure, PVR, pulmonary vascular resistance; WU, Wood units; BR, breathing reserve; MVV, maximal voluntary ventilation; VO_2_, oxygen uptake; RER, respiratory exchange ratio; VE, ventilation; VCO_2_, carbon dioxide production; P_ET_CO_2_, partial pressure of end-tidal CO_2_; AT, anaerobic threshold, ΔSO_2_, difference in oxygen saturation between peak exercise and rest; PaO_2_, arterial partial pressure of oxygen; A-a, alveolar-arterial gradient; V_D_/V_T_, dead-space to tidal volume ratio.

**Table 3:** CT and MRI data

Variable	Group 1 mPAP≤20 mmHg n=15	Group 2 mPAP>20 mmHg and PVR >2-≤3 WU n=15	Group 3 mPAP>20 mmHg and PVR>3 WU n=20	Group 4 “Unclassified” mPAP>20mHg and PVR<2WU n=18	p value
*Mosaicism*					
None or mild	13 (93%)	10 (71%)	16 (94%)	16 (89%)	
Moderate or severe	1 (7%)	4 (29%)	1 (6%)	2 (11%)	0.228
*Scarring*					
Yes	10 (71%)	9 (64%)	13 (76%)	10 (56%)	
No	4 (29%)	5 (36%)	4 (24%)	8 (44%)	0.590
*Clot distribution*					
Proximal	1 (7%)	1 (7%)	1 (6%)	4 (22%)	
Segmental	13 (93%)	11 (79%)	15 (88%)	14 (78%)	
Distal	0 (0%)	2 (14%)	1 (6%)	0 (0%)	0.290
Number of segments involved	9 (+/-4)	9 (+/-4)	8 (+/-5)	8 (+/-4)	0.822
MRI RVEF (%)	52 (+/-13)	57 (+/-8)	46 (+/-12)	53 (+/-6.5)	0.071
RV-PA Coupling	1.07 (+/-0.6)	1.37 (+/-0.3)	0.97 (+/-0.4)	1.12 (+/-0.3)	0.228

mPAP, mean pulmonary artery pressure; PVR, pulmonary vascular resistance; WU, Wood units; MRI, magnetic resonance imaging, RVEF, right ventricular ejection fraction; RV-PA coupling, right ventricular-pulmonary arterial coupling.

**Table 4:** Repeat Cardiopulmonary Exercise Testing (n=19)

Variables	Baseline CPET	Repeat CPET	p value
Work (%pred)	85.4 (+/-26)	83.4 (+/-25)	0.806
BR (% of MVV)	28.2 (+/-13.9)	30.5 (+/-15.7)	0.634
Peak VO_2_ (ml/kg/min)	19.3 (+/-5.8)	19.1 (+/-4.6)	0.907
Peak VO_2_ (% pred.)	75.9 (+/-19)	76.5 (+/-18.4)	0.928
RER peak	1.1 (+/-0.1)	1.1 (+/-0.1)	0.683
VO_2_/work	8.9 (+/-1.4)	9.2 (+/-1.2)	0.557
Peak O_2_ pulse (% pred.)	89.6 (+/-18.7)	85.4 (+/-17.8)	0.487
VE/VCO_2_ nadir	36.9 (+/-6.9)	35.7 (+/-6.5)	0.587
VE/VCO_2_ slope	39.3 (+/-8.1)	39.3 (+/-8.6)	0.981
P_ET_CO_2_ at AT (kPa)	3.8 (+/-0.6)	4.1 (+/-0.8)	0.309
ΔSO_2_ (%)	-3.4 (+/3.7)	-3.4 (+/-3.4)	1
Peak PaO_2_	10.4 (+/-2.3)	11.4 (+/-2.9)	0.38
Peak lactate (mmol/L)	7.2 (+/-2)	7.2 (+/-2)	0.937
A-a gradient	5.3 (+/-1.9)	4.2 (+/-2.3)	0.232
V_D_/V_T_	0.4 (+/-0.1)	0.3 (+/-0.2)	0.116

BR, breathing reserve; MVV, maximal voluntary ventilation; VO_2_, oxygen uptake; RER, respiratory exchange ratio; VE, ventilation; VCO_2_, carbon dioxide production; P_ET_CO_2_, partial pressure of end-tidal CO_2_; AT, anaerobic threshold; ΔSO_2_, difference in oxygen saturation between peak exercise and rest; PaO_2_, arterial partial pressure of oxygen; A-a, alveolar-arterial gradient; V_D_/V_T_, dead-space to tidal volume ratio.
