# Supplementary material for: Do Haemodynamic Definitions of Chronic Thromboembolic Pulmonary Hypertension Distinguish between Distinct Phenotypes of Chronic Thromboembolic Pulmonary Disease?
Source: Ann Am Thorac Soc. Author manuscript; Available in PMC 2025 Mar 1. (PMC7617186; doi:10.1513/AnnalsATS.202405-524OC)
Supplement: Supplementary Materials [file EMS199554-supplement-Supplementary_Materials.docx]

**SUPPLEMANTARY MATERIAL**

**Table S1: Logistic regression analysis: dependent variable VE/VCO_2_>36.**

| **Independent variable** | **Odds ratio** | **95% Confidence Interval** | **p value** |
| --- | --- | --- | --- |
| mPAP | 1.02 | 0.96-1.08 | 0.55 |
| Cardiac output | 0.88 | 0.59-1.32 | 0.527 |
| Pulmonary vascular resistance | 1.42 | 0.95-2.13 | 0.084 |
| NT-proBNP | 1.005 | 1-1.01 | 0.068 |
| Number of segments involved | 1.03 | 0.91-1.15 | 0.666 |
| RVEF% | 1.05 | 0.99-1.11 | 0.096 |

mPAP, mean pulmonary artery pressure; NT-proBNP, N-terminal pro-Brain Natriuretic Peptide; RVEF, right ventricular ejection fraction.

**Table S2: Logistic regression analysis: dependent variable peak O_2_ pulse<80% pred.**

| **Independent variable** | **Odds ratio** | **95% Confidence Interval** | **p value** |
| --- | --- | --- | --- |
| mPAP | 0.95 | 0.9-1.009 | 0.098 |
| Cardiac output | 0.79 | 0.5-1.25 | 0.311 |
| Pulmonary vascular resistance | 0.76 | 0.56-1.04 | 0.091 |
| NT-proBNP | 1 | 1-1.002 | 0.576 |
| Number of segments involved | 1.07 | 0.94-1.22 | 0.29 |
| RVEF% | 1.05 | 0.99-1.11 | 0.102 |

mPAP, mean pulmonary artery pressure; NT-proBNP, N-terminal pro-Brain Natriuretic Peptide; RVEF, right ventricular ejection fraction.

**Cardiopulmonary Exercise Protocol**

Each test was performed in three stages: 3 min of rest, 3 min of unloaded pedaling, and a progressive ramp increase in workload to maximum exercise, with the ramp rate estimated to result in a work phase of 8 to 12 min. Patients wore a face mask. Systemic blood pressure was measured with a sphygmomanometer, finger oxygen saturation was measured with a pulse oximeter, and a 12-lead electrocardiogram was continuously recorded. Breath-by-breath measurements included oxygen uptake (VO_2_), carbon dioxide production (VCO_2_), and ventilation (VE). An arterial blood sample was drawn just after termination of exercise for measurement of arterial partial pressure of oxygen (PaO_2_), arterial partial pressure of carbon dioxide (PaCO_2_) and lactate level. The best estimate of anaerobic threshold (AT) was calculated manually using a combination of the V-slope method and ventilatory equivalents for oxygen. VE/VCO_2_ slope was obtained by linear regression analysis of the relationship between VE and VCO_2_ during exercise prior to the respiratory compensation point. VE/VCO_2_was also measured as ventilatory equivalent for CO_2_at its lowest value (nadir) over a 30-second average, reflecting the highest degree of ventilatory efficiency for each patient.

**Equations**

Breathing reserve (BR) was calculated as MVV - peak ventilation, where MVV is maximal voluntary ventilation, and was calculated as 40xFEV1.

Physiologic dead space was (V_D_/V_T_) was measured using the Bohr equation:V_D_/V_T_ = (PaCO_2_ - P_E_CO_2_)/PaCO_2_, where P_E_CO_2_ is the measured mixed expired partial pressure of CO_2_.
